# Transapical aortic perfusion using a deep hypothermic procedure to prevent dissecting lung injury during re-do thoracoabdominal aortic aneurysm surgery

**DOI:** 10.1186/s13019-017-0601-9

**Published:** 2017-05-19

**Authors:** Yuya Kise, Yukio Kuniyoshi, Mizuki Ando, Hitoshi Inafuku, Takaaki Nagano, Satoshi Yamashiro

**Affiliations:** Department of Thoracic and Cardiovascular Surgery, Graduate School of Medicine, University of the Ryukyus, Ryukyus, 207 Uehara, Nishihara, Okinawa 903-0215 Japan

**Keywords:** Transapical aortic perfusion, Thoracoabdominal aortic aneurysm, Hypothermia, Lung injury

## Abstract

**Background:**

Avoiding various complications is a challenge during re-do thoracoabdominal aneurysm surgery.

**Case presentation:**

A 56-year-old man had undergone surgery for type I aortic dissection four times. The residual thoracoabdominal aortic aneurysm that had severe adhesions to lung parenchyma was resected. Since the proximal anastomotic site was buried in lung parenchyma, deep hypothermia was essential to avoid lung dissection and to protect the spinal cord during the proximal anastomosis. The deep hypothermia was induced with bilateral infusion of cardiopulmonary bypass by femoral artery cannulation for the lower body and by transapical cannulation for the upper body because of easy access. There was no hemorrhagic tendency after deep hypothermic bypass. The patient was discharged uneventfully.

**Conclusions:**

For upper body perfusion, transapical aortic cannulation was a simple and effective procedure during left thoracotomy.

## Background

In thoracoabdominal aortic aneurysm (TAAA) surgery with severe lung adhesions caused by repeat operations, lung dissection followed by lung injuries causes postoperative respiratory failure. To avoid lung dissection and approach the proximal anastomotic site, deep hypothermic cardiopulmonary bypass (CPB) is essential, because the procedure of approaching the proximal anastomosis is time consuming and may cause spinal cord ischemic injury under normothermic CPB.

In cases with previous ascending aortic surgery, the approach for the CPB infusion site is troublesome. Transapical aortic cannulation is a novel and easy procedure for antegrade perfusion, and it could work for selective cardiac and cerebral perfusion by clamping the aorta distal to the left subclavian artery.

Deep hypothermic CPB by transapical aortic cannulation might be useful in cases of a frequently operated, extended TAAA to avoid unnecessary dissection of the lung to find the proximal anastomosis and proximal aorta for cannulation.

## Case presentation

**Fig. 1:**
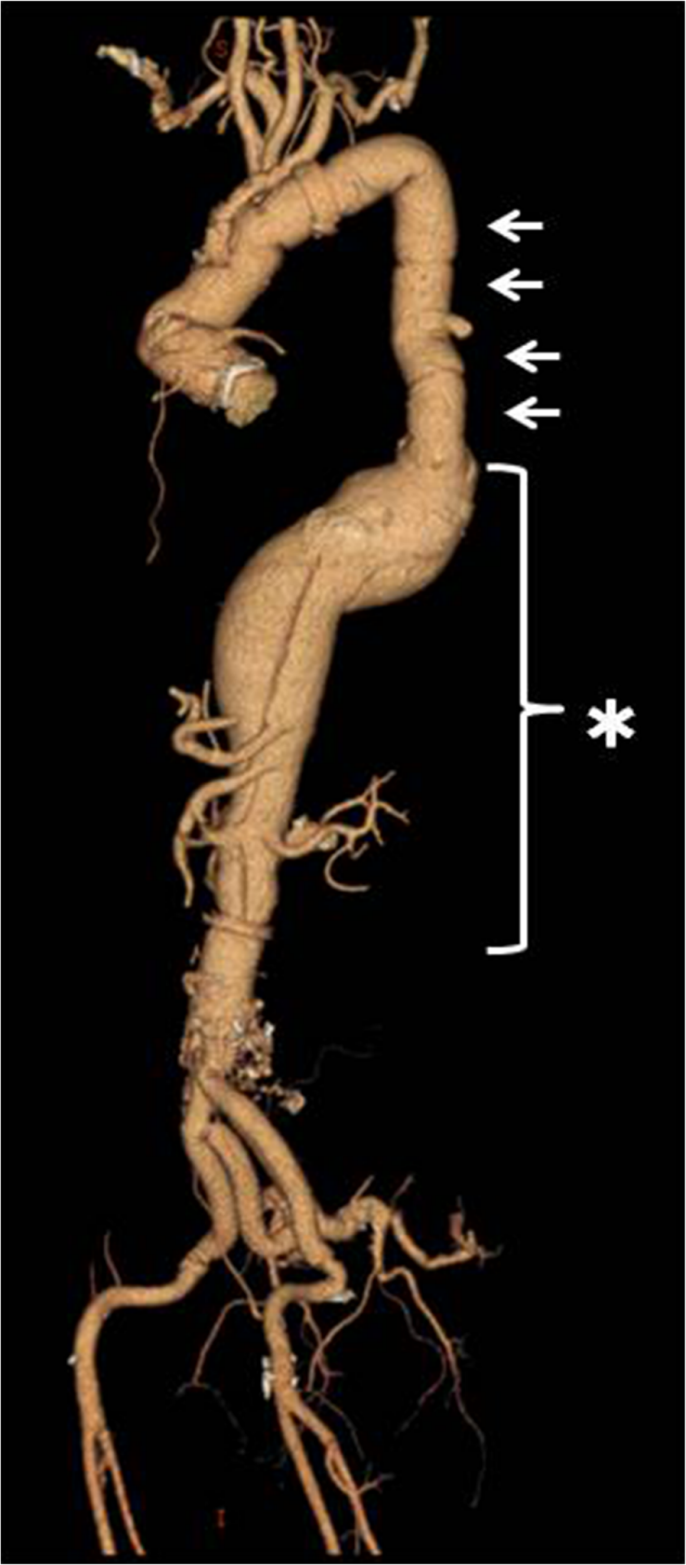
Preoperative three-dimensional CT showing a chronic dissectional thoracoabdominal aortic aneurysm from the descending aorta (Th7 level) to the infrarenal aorta (*asterisk*). The severe adhesions between the descending prosthetic graft and the *left* lung are predictable (*arrow*)

The patient was a 56-year-old man who had undergone four surgical procedures for DeBakey type I dissection from 2002 to 2013: (1) graft replacement of the ascending aorta to the aortic arch (2002); (2) graft replacement of the proximal descending aorta to the Th7 level (2003); (3) Bentall operation (with a bioprosthetic valve) (2012); and (4) graft replacement of the infrarenal abdominal aorta to the bilateral external iliac arteries (2013). The remaining aorta was a TAAA from TH7 to the abdominal aorta distal to the renal artery, with a diameter of 52 mm. In September 2014, recurrent aortic dissection occurred in the remaining TAAA (Fig. 1). After the symptoms of dissection subsided, the TAAA operation was performed.

## Operative procedure

**Fig. 2:**
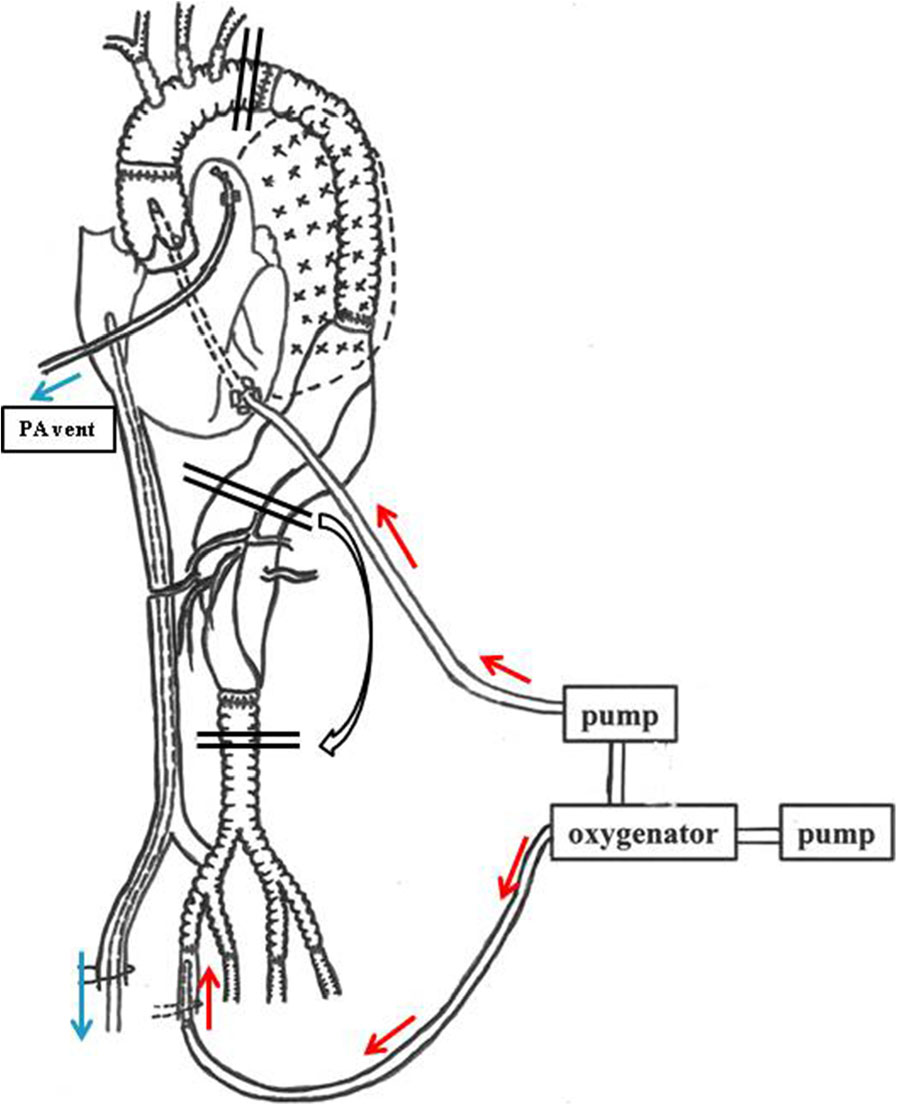
Schema of perfusion technique. Transapical aortic perfusion and right femoral perfusion were done respectively. After core cooling, selective perfusion of the upper and lower body was continued under cross-clamping of the site just distal to the *left* subclavian artery and diaphragmatic site. After the proximal anastomosis, the distal clamp was moved to the distal portion of the TAAA

On a pre-operative computed tomography scan, the left side of the segmental artery at the Th11 level was identified as the Adamkiewicz artery (AKA). As for the surgical procedure, a left thoracotomy by Stoney incision was performed with the patient in the right lateral decubitus position, and the retroperitoneal space was approached. The infradiaphragmatic aortic aneurysm was exposed, but the intrathoracic aortic aneurysm could not be approached because of severe adhesions of the graft prosthesis and lung parenchyma, except for the apical portion of the left lung where there was not much adhesion to the graft prosthesis replaced in the past operation. There were also severe adhesions of the proximal thoracic aorta. As a cannulation site for upper body perfusion, the left ventricular (LV) apex was easily approached for cannulation in the ascending aorta. Heparin was administered at 300 units/kg, and then transapical aortic cannulation (EZ Glide 24Fr; Edwards Lifescience, Irvine, CA) was set with monitoring by transesophageal echocardiography (TEE). The right femoral artery (FEM II 16Fr; Edwards Lifescience) was cannulated for perfusion of the lower body. CPB by drainage from a long cannula from the right femoral vein to the right atrium was started, with central cooling to 16 °C. After 30 min of sufficient core cooling, the aortic aneurysm that adhered in the thorax was excluded by clamping at just distal to the left subclavian artery and the diaphragmatic site. Cardiac and cerebral selective perfusion was established. The flow rates to the transapical cannula and the right femoral artery were regulated by pressure monitoring of the right radial artery and the left femoral artery, respectively (Fig. 2). To avoid spinal cord ischemia, motor evoked potentials (MEPs) were monitored continuously; their amplitude decreased with lowering of the body temperature, and it was zero at less than 25 °C. The target of the proximal site, the distal edge of the thoracic descending prosthetic graft, was reached by incising the aneurysmal wall where it had not adhered to the lung parenchyma, and it was prepared by an encircled incision for anastomosis with a 26-mm, size 4-branch graft for TAAA (J-Graft SHIELD NEO; Japan Lifeline, Tokyo, Japan). After anastomosis, the proximal clamp forceps was released to confirm hemostasis of this anastomotic site. Sequentially, the distal clamping was moved to the distal portion of the TAAA, with reconstruction of the AKA, intercostal artery, and four major abdominal branches. Finally, the graft-graft anastomosis was done. Rewarming was started after reconstruction of the AKA and intercostal artery, and MEPs returned at a temperature greater than 25 °C. The data related to CPB were as follows. The time required from when the aortic cross-clamping until the proximal site anastomosis was completed was 50 min, and the time from when aortic cross-clamping was initiated until AKA and segmental artery reconstruction was 105 min. There was no postoperative pulmonary hemorrhage. The patient was extubated on Day 4, and he was discharged on Day 26, able to walk independently and free of paraplegia.

## Discussion

Multiple surgeries are usual for an extended aortic aneurysm, but this approach involves high operative risk, especially at re-do thoracic aortic surgery. Several postoperative morbidities, including spinal cord ischemic injury, respiratory failure, multiple organ failure, and others, have been reported. In particular, lung injury may be caused by lung dissection-induced postoperative respiratory failure, or graft infection may occur with leakage from the lung, which can be lethal. To prevent such complications, deep hypothermic technique was applied. Hypothermic CPB at TAAA surgery has been reported as a possible adjunct for organ protection by some authors [1–3]. However, there are a few reports of preventing postoperative lung complications by hypothermic CPB [3]. In this case, hypothermic CPB was used for this purpose.

However, when only deep hypothermic circulatory arrest is used, the time for proximal anastomosis is limited to approximately 30 min, and sufficient protection of the brain and myocardium is a concern if the procedure must be prolonged. Recent reports of TAAA surgery using deep hypothermic circulatory arrest have shown relatively good results in terms of the frequency of spinal cord ischemic injury (1.3 to 5%), but because the incidence of stroke is from 4 to 7%, and the incidence of postoperative low output syndrome is from 1.2 to 14%, protection of the brain and myocardium is still problematic [1, 2, 4, 5].

Continuing to use the advantages of deep hypothermic technique while also performing selective perfusion of the upper and lower body is believed to be one countermeasure to ensure cerebral and coronary perfusion and spinal cord blood supply via the collateral network from the vertebral artery, subclavian artery, internal thoracic artery, and internal iliac artery [6]. In the 1990s, the utility of selective upper and lower body blood perfusion was reported during thoracoabdominal aortic aneurysm surgery [7]. Later, a technique for changing the blood temperature and the perfusion rate selectively in the upper and lower body depending on the purpose of the protected organs was also reported [8]. The ascending aorta and thoracic descending aorta are frequently selected as the cannulation route for perfusing the upper body, but this route is not appropriate in patients with profound atherosclerotic changes, such as a shaggy and calcified aorta [9]. Shiiya et al. reported using antegrade perfusion by transapical aortic cannulation to prevent debris scattering due to retrograde perfusion from the femoral artery in a patient undergoing thoracoabdominal aortic repair by left thoracotomy [7], and Takemura et al. reported the usefulness of transapical cannulation in preventing malperfusion during a deep hypothermic procedure to repair an acute traumatic descending aortic rupture [10]. Although antegrade perfusion via the axillary artery is safe and possible [11], it is difficult to ensure the visual field during a left thoracotomy procedure.

Transapical aortic cannulation during left thoracotomy has the advantage of enabling the definitive placement of the perfusion cannula in the ascending aorta and enabling antegrade perfusion, without dislocating the heart. In this patient, too, a prosthetic graft replacement had previously been performed on the ascending to descending aorta, and direct cannulation to the graft replacement site was expected to be difficult. Transapical cannula insertion where the apex is located in front of the operating field was performed even more safely under TEE guidance, and when removing the cannula, hemostasis to the apex could be done without difficulty.

## Conclusion

During re-do thoracoabdominal aortic aneurysm repair, upper body perfusion by transapical aortic cannulation might be a safe alternative technique without exposure of the proximal thoracic aorta for cannulation.

## Abbreviations

AKA: Adamkiewicz artery
CPB: Cardiopulmonary bypass
MEPs: Motor evoked potentials
TAAA: Thoracoabdominal aortic aneurysm
TEE: Transesophageal echocardiography
